# NRF2, a Transcription Factor for Stress Response and Beyond

**DOI:** 10.3390/ijms21134777

**Published:** 2020-07-06

**Authors:** Feng He, Xiaoli Ru, Tao Wen

**Affiliations:** 1 Laboratory of Gene Regulation and Signal Transduction, Departments of Pharmacology, University of California San Diego, 9500 Gilman Drive, La Jolla, CA 92093, USA; 2 Department of Gynecology and Obstetrics, Beijing Chao-Yang Hospital, Capital Medical University, Beijing 100020, China; 18801350216@163.com; 3 Medical Research Center, Beijing Chao-Yang Hospital, Capital Medical University, Beijing 100020, China

**Keywords:** NRF2, metabolism, UPR, oxidative stress, inflammation, autophagy, proteostasis, transcription factor

## Abstract

Nuclear factor erythroid 2-related factor 2 (NRF2) is a transcription factor that regulates the cellular defense against toxic and oxidative insults through the expression of genes involved in oxidative stress response and drug detoxification. NRF2 activation renders cells resistant to chemical carcinogens and inflammatory challenges. In addition to antioxidant responses, NRF2 is involved in many other cellular processes, including metabolism and inflammation, and its functions are beyond the originally envisioned. NRF2 activity is tightly regulated through a complex transcriptional and post-translational network that enables it to orchestrate the cell’s response and adaptation to various pathological stressors for the homeostasis maintenance. Elevated or decreased NRF2 activity by pharmacological and genetic manipulations of NRF2 activation is associated with many metabolism- or inflammation-related diseases. Emerging evidence shows that NRF2 lies at the center of a complex regulatory network and establishes NRF2 as a truly pleiotropic transcription factor. Here we summarize the complex regulatory network of NRF2 activity and its roles in metabolic reprogramming, unfolded protein response, proteostasis, autophagy, mitochondrial biogenesis, inflammation, and immunity.

## 1. Introduction

**Figure 1:**
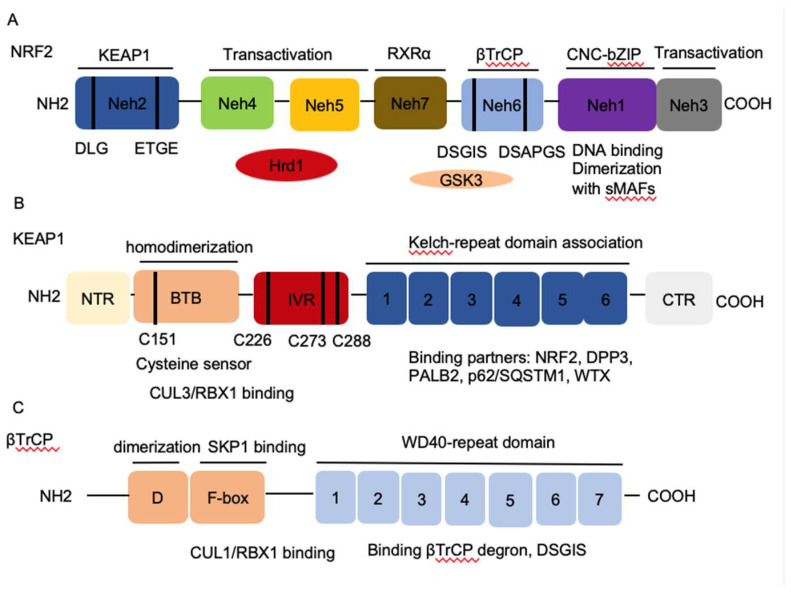
The architecture of Nuclear factor erythroid 2-related factor 2 (NRF2), Kelch-like-ECH-associated protein 1 (KEAP1), and β-transducin repeat-containing protein (βTrCP). (**A**) NRF2 contains seven conserved NRF2-ECH homology NRF2-ECH homology (Neh) domains, Neh1-Neh7. Neh1 contains a basic leucine zipper (bZip) motif, where the basic region is responsible for DNA binding and the Zip dimerizes with other binding partners such as sMAFs. Neh2 contains ETGE and DLG motifs, which are required for the interaction with KEAP1 and subsequent KEAP1-mediated proteasomal degradation. Neh3, 4 and 5 domains are transactivation domains of NRF2. Neh4 and 5 domains also interact with HRD1 that mediates NRF2 degradation. Neh6 contains two βTrCP degrons DSGIS and DSAPGS that are responsible for the β-TrCP mediated proteasomal degradation. (**B**) KEAP1 contains five domains, amino terminal region (NTR), a broad complex, tramtrack, bric-a-brac (BTB) domain, an intervening region (IVR), six Kelch domains, and the C-terminal region (CTR). The Kelch domain and CTR mediate the interaction with NRF2, p62, DPP3, WTX, and PALB2 that contains ETGE motifs. The BTB domain homodimerizes with KEAP1 and contributes to the interaction of IVR with Cul3/RBX1 complex. Several functional important cysteine residues (C151, C226, C273 and C278) that sense reactive oxygen species (ROS) and electrophiles and modulate KEAP1-NRF2 interaction. (**C**) βTrCP has three domains, dimerization domain (D) that forms homo- and heterodimers between βTrCP1 and βTrCP2, the F-box that recruits SKP1 for the binding of CUL1/RBX1 complex, and the WD40 repeat domain that binds βTrCP degrons DSGIS and DSAPGS in NRF2. βTrCP, β-transducing repeat-containing protein; CUL3, Cullin3; RBX1, RING-box protein; WD40, WD Repeat protein 40; RXRα, retinoic X receptor alpha; DPP3, dipeptidyl peptidase 3; WTX, Wilms tumor gene on X chromosome; PALB2, Partner and Localizer of BRCA2; GSK3, Glycogen synthase kinase-3; SKP1, S-phase kinase-associated protein-1.

Homeostasis is key to organismal health and survival. Environmental stress is ubiquitous and unavoidable to all living beings and threatens to disrupt cell functions. Organisms respond and adapt to stresses through defined regulatory mechanisms. The transcription factor nuclear factor erythroid 2-related factor 2 (NRF2) is best known as one of the main orchestrators of the cellular xenobiotic and oxidative stress response. NRF2, encoded by the gene nuclear factor, erythroid 2 like 2 (*NFE2L2*), belongs to the Cap’n’Collar (CNC) subfamily of basic leucine zipper (bZIP) transcription factors, which comprises nuclear factor erythroid-derived 2 (NFE2) and NRF1, NRF2, and NRF3 [1]. NRF2 possesses seven conserved NRF2-ECH homology (Neh) domains with different functions to control NRF2 transcriptional activity (Figure 1A). The bZip in the Neh1 domain heterodimerizes with small musculoaponeurotic fibrosarcoma proteins (sMAF) K, G, and F as well as other bZip proteins to recognize antioxidant response elements (ARE) for activation of gene transcription, whereas the Neh2 domain contains ETGE and DLG motifs that specifically interact with Kelch domain of Kelch-like-ECH-associated protein 1 (KEAP1) to mediate NRF2 ubiquitination and degradation (Figure 1B) [2]. The Neh3-5 domains function as transcriptional activation domains by binding to various components of the transcriptional machinery [3]. Neh6 domain contains two redox-independent degrons DSGIS and DSAPGS that bind to E3 ubiquitin ligase β-transducin repeat-containing protein (βTrCP), which mediates NRF2 degradation in oxidatively stressed cells (Figure 1C) [4]. Neh7 domain mediates interaction with retinoic X receptor alpha (RXRα), which represses NRF2 activity [5]. These domains modulate NRF2 stability and transcriptional activation of its target genes at multiple levels, including transcriptional and post-transcriptional and post-translational regulation in response to various insults. Recent studies have identified new NRF2 target genes and revealed several new functions of NRF2 that go beyond its redox-regulating capacities, including regulation of inflammation, autophagy, metabolism, proteostasis, and unfolded protein response (UPR), particularly in the context of carcinogenesis. NRF2 has become a prime subject of extensive research involving inflammation, metabolism, cancer prevention and treatment, and its functions are more far-reaching than originally envisioned. Understanding the regulation of NRF2 activity and its new emerging functions presents new challenges but also new opportunities for targeting NRF2 in cancer.

## 2. Regulation of NRF2 Activity

### 2.1. Regulation of NRF2 Protein Stability

**Figure 2:**
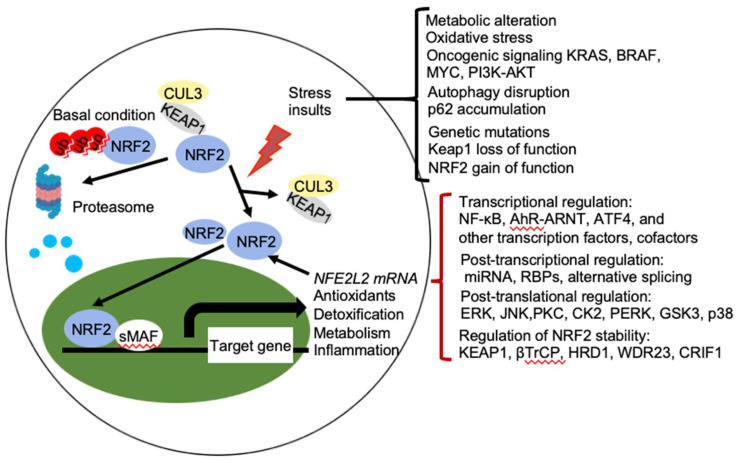
Regulation of NRF2 activity. Under basal conditions, the amount of NRF2 is low due to its continuous sequestration by KEAP1 and subsequent proteasomal degradation. Under stressed condition, the cellular NRF2 amount is temporarily or constitutively increased upon exposure to toxicants and ROS, oncogenic signaling, genetic mutations, autophagy disruption, or metabolic alteration, which disrupt the KEAP1-NRF2 complex and lead to activation of NRF2. Activated NRF2 accumulates in the nucleus, where it interacts with other transcription factors and cofactors to regulate transcription of its target genes, which encoding proteins involved in the antioxidants, detoxification, metabolism, and inflammation. NRF2 also regulates its own *NFE2L2* mRNA transcription. Activity of NRF2 is modulated at multiple levels, including transcriptional regulation (NF-κB, AhR-ARNT, ATF4, and other transcription factors, cofactors), post-transcriptional regulation (miRNA, RBPs, alternative splicing), post-translational regulation (ERK, JNK, PKC, CK2, PERK, GSK3, p38), and regulation of NRF2 stability (KEAP1, βTrCP, HRD1, WDR23, CRIF1). AhR, aryl hydrocarbon receptor; ARNT, AHR nuclear translocator; NF-κB, nuclear factor-κB; PI3K, phopshoinositide 3-kinase; PKC, protein kinase C; ERG, extracellular signal-regulated protein kinases; JNK, c-jun *N*-terminal kinase; PERK, protein kinase R (PKR)-like endoplasmic reticulum kinase; CK2, casein kinase 2; WDR23, WD40-repeat protein 23; CRIF1, CR6-interacting Factor 1; ATF4, activating transcription factor.

NRF2 abundance within the cell is tightly regulated and is mainly controlled by four E3 ubiquitin ligase complexes-mediated ubiquitylation and proteasomal degradation: KEAP1-Cullin (CUL) 3-RING-box protein (RBX)1, βTrCP-S-phase kinase-associated protein-1 (SKP1)-CUL1-RBX1, WD Repeat protein (WDR), 3-CUL4-damaged DNA binding protein (DDB) 1, and HRD1 (also called Synoviolin) under different conditions. NRF2 is expressed in all cell types and its basal protein levels are usually low in unstressed conditions mainly due to KEAP1 mediated proteasomal degradation (Figure 2). KEAP1 is a redox-regulated adaptor for the CUL3- RBX1 ubiquitin ligase complex and it binds NRF2 through its C-terminal Kelch domain, which interacts with the DLG and ETGE motifs in the Neh2 domain of NRF2, resulting in ubiquitination of NRF2 in the cytoplasm and degradation by the 26 S proteasome [3]. This constitutive degradation of NRF2 ensures that only the basal expression of NRF2 target genes are maintained for housekeeping functions. During oxidative stress, electrophiles and reactive oxygen species (ROS) react with sensor cysteines of KEAP1, including cysteine 151 (C151), C273, and C288, to allow NRF2 to escape from KEAP1 mediated degradation [6,7]. As a result, newly synthesized NRF2 accumulates in the nucleus and activates the expression of cytoprotective genes [3,8]. In addition, KEAP1 dependent but cysteine independent mechanisms were reported to stabilize NRF2 by interfering with formation of the NRF2-KEAP1 complex. Autophagy cargo-adaptor p62/sequestosome 1 (SQSTM1) [9,10,11], dipeptidyl peptidase 3 (DPP3) [12], Wilms tumor gene on X chromosome (WTX) [13], and Partner and Localizer of BRCA2 (PALB2) [14] all contain KEAP1-interacting region (KIR)-like ETGE motifs and thus competes with NRF2 for KEAP1 binding, resulting in KEAP1 sequestration and NRF2 stabilization. p21Cip1/WAF1 [15] and Breast Cancer Type 1 Susceptibility Protein (BRCA1) [16] can also directly interact with the ETGE motif of NRF2, thus competes with KEAP1 for NRF2 binding and stabilizes NRF2. Furthermore, the acetyltransferase p300 stabilizes NRF2 and increases its nuclear localization by interfering KEAP1-NRF2 interaction [17].

In contrast to KEAP1-CUL3-RBX1 mediated degradation under basal conditions, βTrCP-SKP1-CUL1-RBX1 and WDR3-CUL4-DDB1 can contribute to NRF2 degradation under both basal and oxidative stress conditions, independent of KEAP1 [18,19,20]. NRF2 contains two motifs, DSGIS and DSAPGS in its Neh6 domain, which can be recognized by the F box of the WD40 substrate adaptor βTrCP. Glycogen synthase kinase-3 (GSK3) phosphorylates the DSGIS motif and increases the affinity of βTrCP for NRF2, thereby stimulating NRF2 ubiquitination and degradation [2,18,19]. GSK3 is inhibited by phosphorylation of an *N*-terminal serine residue (Ser9 in GSK-3β and Ser21 GSK-3α, respectively) by protein kinase B (PKB)/AKT. The signaling that activates upstream phosphoinositide 3-kinase (PI3K)-PKB/AKT, including growth factors, mTOR, and oncogenic RAS signaling, inhibits GSK3 and thereby stabilizes NRF2 by suppressing βTrCP-mediated degradation [19]. WDR23 is a WD40-repeat protein and it binds the DIDLID sequence within the Neh2 domain of NRF2 to regulate NRF2 ubiquitination and degradation [20]. HRD1 is an endoplasmic reticulum (ER) membrane-associated E3 ubiquitin ligase involved in ER-associated degradation (ERAD). Under ER stress conditions, HRD1 is induced and binds to the Neh 4-5 domains of NRF2 that mediate NRF2 ubiquitylation and degradation in cirrhotic livers [21]. In addition, CR6-interacting Factor 1 (CRIF1), also known as growth arrest and DNA damage-inducible proteins-interacting protein 1 (GADD45GIP1), interacts with Neh2 domain and the C-terminal region containing Neh1 and Neh3 of NRF2 to promote NRF2 ubiquitylation and degradation in a redox-independent manner [22]. Interestingly, KEAP1-mediated NRF2 ubiquitination and degradation mainly act in the cytoplasm, whereas KEAP-1 independent NRF2 stability regulation is both cytoplasmic and nuclear, which contributes to termination of NRF2-mediated transcriptional response [20,22,23]. Furthermore, posttranslational modifications of NRF2 contribute to the changes in its binding partners and cellular localization that regulate NRF2 stability. Extracellular signal-regulated protein kinases (ERK) [24], c-jun *N*-terminal kinase (JNK) [24], PI3K-AKT [19], protein kinase C (PKC) [25], casein kinase 2 (CK2) [26], and protein kinase R (PKR)-like endoplasmic reticulum kinase (PERK) [27] were reported to mediate phosphorylation of NRF2 and increase its stability and subsequent transcriptional activity, whereas p38 and GSK3-mediated phosphorylation of NRF2 decreases NRF2 stability [19]. In hepatocellular carcinoma triggered by MYC and KEAP1 inactivation, fructosamine-3-kinase (FN3K), a kinase that triggers protein de-glycation, mediates NRF2 de-glycation that stabilizes NRF2 and executes its oncogenic function [28].

### 2.2. Regulation of NRF2 Transcription

Transcription factor NRF2 binds to ARE in the promoter region of many cell defense genes to activate their transcription. *NFE2L2* gene contains ARE within its promoter region that renders NRF2 the ability to directly activate its own transcription, providing a positive feedback mechanism to amplify NRF2 effects [29]. In addition, *NFE2L2* transcription is regulated by several transcription factors, including aryl hydrocarbon receptor (AhR) [30] and nuclear factor (NF)-κB [31]. The *NFE2L2* gene contains one xenobiotic-responsive element (XRE)-like element at position -712 of the promoter region and two additional XRE-like elements located at +755 and +850 that are activated by the transcription factor aryl hydrocarbon receptor (AHR) [30]. When activated by ligands, such as TCDD (2,3,7,8-tetrachlorodibenzo-p-dioxin), AHR dimerizes with AHR nuclear translocator (ARNT) to bind to the XRE of *NFE2L2* and activates NRF2 transcription [30]. In addition, *NFE2L2* promoter contains an NF-κB binding site, which allows it to be regulated by NF-κB [32]. Lipopolysaccharide (LPS) treatment in human monocytes induced *NFE2L2* transcription [33]. Constitutive NF-κB activation mediates upregulation of *NFE2L2* gene and contributes to high basal NRF2 activity that renders resistance to chemotherapy in acute myeloid leukemia (AML) [32]. Oncogenic signaling pathways including oncogenic oncogenes K-RAS(G12D), B-RAF (V619E) and MYC (ERT2) [34], the PI3K-AKT pathway [35], and the Notch signaling pathway [36], have been reported to augment *NFE2L2* transcription to stably elevate the basal NRF2 antioxidant program and support its pro-tumorigenic effects. Some hypermethylation or single nucleotide polymorphisms (SNPs) of the *NFE2L2* promoter region decreases NRF2 mRNA expression [26,37,38].

### 2.3. Post-Transcriptional Regulation of NRF2

microRNAs (miRNAs) are endogenous single-stranded, noncoding RNAs with an average 22 nucleotides in length that repress gene expression by sequence-specific binding with mRNA molecules and subsequent inhibition of protein translation and destabilization of mRNA [39]. miRNAs that have been shown to be involved in the regulation of NRF2 include miR-144 [40], miR-28 [41], miR-34 [42], miR-93 [43], and miR-153 [44]. Increased miR-144 is associated with reduced NRF2 transcriptional activity and impaired oxidative stress tolerance in erythroid cells, which is associated with the sickle cell disease [40]. miR-34 expression is increased in aging rat liver, which targets NRF2 and downstream oxidative stress defense protein Mgst1s to inhibit their expression [42]. Expression of miR-28 [41], miR-93 [43], and miR-153 [44] in breast cancer cells were reported to facilitate the degradation of NRF2 mRNA. In neuroblastoma cells, miR-153, miR-27a, miR142-5p, and miR-144 directly target 3′-untranslated region (UTR) of *NFE2L2* and changes in these miRNAs either individually or as a group could result in inefficient transactivating ability of NRF2 [45]. However, validation in physiological and pathological conditions is still lacking.

In contrast to miRNAs, RNA-binding proteins (RBPs) are typically considered as proteins that bind RNA through one or multiple globular RNA-binding domains (RBDs) and change the fate or function of the bound RNAs [46]. Two of RBPs, HuR and AUF1 binds to the 3′-UTR of *NFE2L2* mRNA and result in the elevated NRF2 activation [47]. HuR enhances *NFE2L2* mRNA maturation and promotes its nuclear export, whereas AUF1 stabilizes *NFE2L2* mRNA [47]. Alternative splicing is another mechanism that can regulate NRF2 activity. Aberrant *NFE2L2* transcript variants lacking exon 2, or exons 2 and 3, have been observed in lung and head and neck cancers and the NRF2 protein isoforms encoded by these splice variants lack the KEAP1 interaction domain, thus resulting in NRF2 stabilization and induction of the NRF2 program [48]. The impact of post-transcriptional regulation of *NFE2L2* on the pathogenesis of disease remains to be evaluated.

### 2.4. Regulation of NRF2 Transcriptional Activation of Its Target Genes

NRF2 activity is tightly regulated. Gene transcription profiles revealed that not all genes in the vicinity of bound NRF2 are transcriptionally regulated by NRF2 binding [49,50]. These genes require the recruitment of both NRF2 and NRF2 binding partners including transcription factors, cofactors, and mediators for a complete activation. sMAF transcription factors, including MAFF, MAFG, and MAFK, binds to NRF2 through their leucine zipper domains to form NRF2-MAF complexes for the recognition of ARE and supports NRF2-mediated transcriptional activation of antioxidant and metabolic genes [51]. The transcription factor BTB and CNC homology 1 (BACH1), which is essential in controlling heme level through the inhibition of heme oxygenase-1 (HO)-1 gene, competes with NRF2-sMAF interaction at ARE sites for transcriptional repression of NRF2 target NAD(P)H:quinone oxidoreductase1 (NQO1) [52]. NRF2-induced HO-1 expression requires inactivation of BACH1 [53]. Activating transcription factor 4 (ATF4) can dimerize with NRF2 at ARE to induce expression of HO-1 [54]. Activator protein 1 (AP-1) subunit c-Jun can dimerize with NRF2 and activate NRF2-induced transcription, while another AP-1 subunit c-Fos can suppress NRF2-induced transcription [55,56].

CREB-binding protein (CBP) and its cofactor p300 can be co-recruited to ARE by NRF2 through its Neh4/5 domains of NRF2 for transcriptional activation [57]. CBP and p300 are histone acetyltransferases to facilitate chromatin decondensation and the recruitment of the transcription machinery [58]. However, ATF3 can compete with CBP for the binding of NRF2 and inhibit the transcription induced by NRF2–CBP complex [59]. Interestingly, ATF3 deletion increases NRF2 degradation through upregulating KEAP1 expression and loss of DJ-1 pathways in human bronchoalveolar epithelial cells [60]. Therefore, ATF3 can either positively or negatively control NRF2 activity. Transcriptional repressor silencing mediator for retinoid and thyroid hormone receptor (SMRT) that mediates histone deacetylation, can interact with NRF2 Neh4/5 domains and inhibit NRF2-induced GSTA2 expression [61].In addition to histone-modifying enzymes, NRF2 interacts with other co-activators of the transcription machinery, such as ATPase subunit of the SWI/SNF chromatin-remodeling complex Brahma-related gene 1 (BRG-1) [62], chromodomain helicase DNA-binding protein 6 (CHD6) [63], receptor-associated co-activator 3 (RAC3) [64], and NAD^+^-dependent histone deacetylase sirtuin 6 (SIRT6) [65], mediator of RNA polymerase II transcription subunit 16 [66] to selectively influence the transcription of NRF2 target genes. However, the functional significance of these interactions has not been extensively elucidated. Nuclear receptors PPARγ [67] and estrogen receptor α (ERα) [68] can directly bind to NRF2 and suppress the NRF2 activity. Interestingly, a gene dose response study analyzing expression changes in livers from *Nfe2l2*-null, wild-type, *Keap1*-knockdown, and *Keap1*-knockout mice showed that the extent of NRF2 transactivation depends on the levels of NRF2 protein (184). Complete understanding of NRF2-mediated gene transactivation requires paying attention to the cooperation or competition with other transcription factors and co-factors at the ARE and ARE-like sites.

## 3. NRF2 Regulates Antioxidant Stress Response and Drug Detoxification

Since NRF2 was first discovered in 1994 as a member of the human CNC-bZIP transcription factor family for the transcriptional stimulation of beta-globin genes [69], over past few decades, many studies have revealed the major role of NRF2 as a transcription factor for antioxidant stress response and drug detoxification. NRF2-induced expression of ARE-containing cytoprotective genes in response to cell stress forms a network of cooperating enzymes involved in phase I, II, and III drug detoxification reaction and elimination of pro-oxidants to maintain cellular homeostasis [70]. Phase I enzymes mediate oxidation, reduction, and hydrolytic reactions of xenobiotics, including NQO1, carbonyl reductases (CBRs), aldo-keto reductases (AKRs), and aldehyde dehydrogenase 1 (ALDH1), and certain cytochrome P450 oxidoreductases cytochrome P450s (CYPs) [71]. Phase II enzymes traditionally refer to the enzymes catalyzing the conjugation reactions, such as glutathione S-transferase (GST), UDP-glucuronosyltransferase (UGT), UDP-glucuronic acid synthesis enzymes, and HO-1 [70,71]. Phase III enzymes transport the conjugated metabolites after Phase II and are mainly drug efflux transporters, such as multidrug resistance-associated proteins (MDR), breast cancer resistant protein (BCRP), ATP-binding cassette g5 (ABCG5) and g8 (ABCG8) [71]. General antioxidant pathways induced by NRF2 include enzymes for the reduced glutathione (GSH) production, utilization, and regeneration. Glutamate-cysteine ligase catalytic (GCLC) and modulator (GCLM) subunits as well as glutathione synthetase (GSS) are the three NRF2 targets involved in the GSH synthesis [23]. The redox cycling enzymes thioredoxin, thioredoxin reductase, sulfiredoxin, peroxiredoxin, glutathione peroxidase, superoxide dismutase 1 (SOD1), and catalase (CAT), and several glutathione S-transferases, which are the enzymes mediating the elimination of reactive oxygen species (ROS), are all NRF2 targets [23]. Glutathione reductase (GR) mediates the reduction of glutathione for the GSH regeneration at the expense of NADPH, which is also a cofactor used in anabolic reactions [23]. These comprehensive cytoprotective proteins encoded by NRF2-target genes are essential for protection against a variety of toxic and oxidative insults and therefore many diseases that have oxidative stress as underlying pathological features, including cardiovascular disease, metabolic syndrome, neuronal degeneration, autoimmune disorders, and cancer.

While searching for genome-wide targets of NRF2, including the generation of *Nfe2l2*-knockout mice [72], *Keap1*-knockdown mice and *Keap1*-knockout mice [73,74], as well as constitutive active NRF2-E79Q-knockin mice [75], many antioxidant response element (ARE) containing genes beyond the conventional antioxidant stress response have been identified. The field of NRF2 is evolving and new functions of NRF2 continue to be discovered, particularly in the pathology of various diseases.

## 4. NRF2 Regulates Metabolic Reprogramming

**Figure 3:**
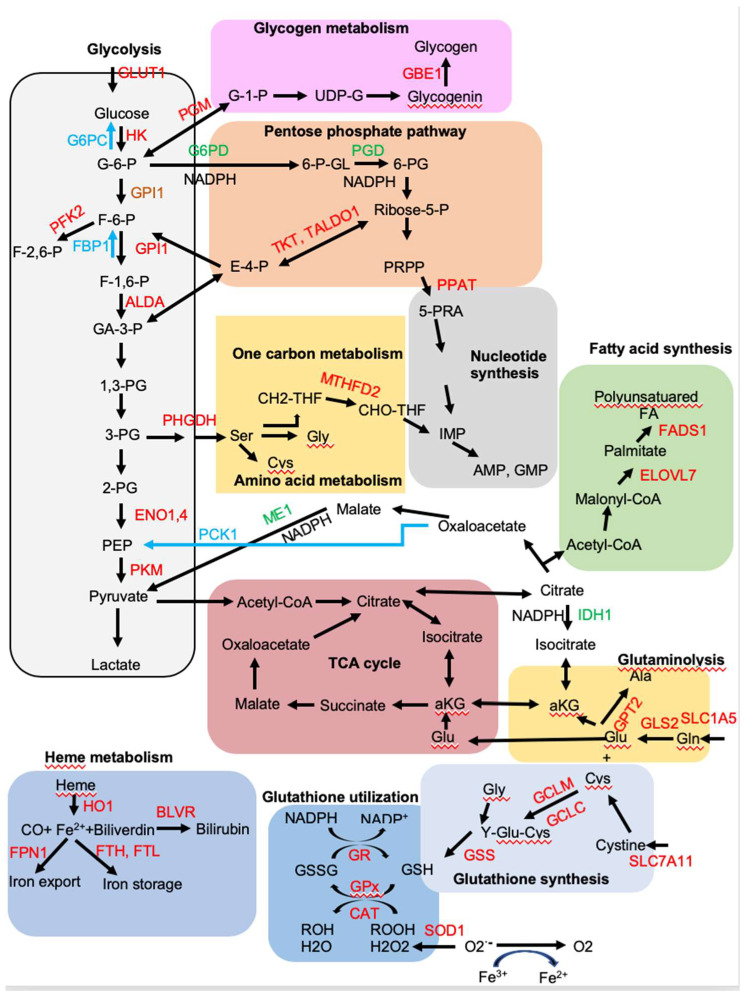
NRF2 regulates metabolism. NRF2 upregulates expression of genes involved in the glycolysis, glycogen metabolism, pentose phosphate pathway, one carbon metabolism, amino acid metabolism, nucleotide biosynthesis, glutaminolysis, fatty acid synthesis, heme metabolism, and glutathione synthesis and utilization (colored in red). NRF2 downregulates the expression of genes involved in the gluconeogenesis (colored in blue). Enzyme reactions that produce NADPH are indicated and colored in green. Enzyme abbreviations: ELOVL7, fatty acid elongase 7; FADS1, fatty acid desaturase 1; G6PD, glucose-6-phosphate dehydrogenase; GCLC, glutamate-cysteine ligase, catalytic subunit; GCLM, glutamate-cysteine ligase, modifier subunit; GLS2, glutaminase 2; GPT2, glutamic pyruvate transaminase; GSS, glutathione synthetase; IDH1, isocitrate dehydrogenase 1; ME1, malic enzyme 1; MTFHD2, methylenetetrahydrofolate dehydrogenase 2; PGD, 6-phosphogluconate dehydrogenase; PHGDH, phosphoglycerate dehydrogenase; PPAT, phosphoribosyl pyrophosphate amidotransferase; TALDO, transaldolase; TKT, transketolase; TXN, thioredoxin; SLC7A11, Solute Carrier Family 7 Member 11; SLC1A5, solute carrier family 1, member 5; HK1, hexokinase 1; GPI1 glucose phosphate isomerase 1; PFK2, 6-phosphofructo-2-kinase; ALDA, aldolase A, fructose-bisphosphate; Eno1,4, enolase 1 and enolase 4; PKM, pyruvate kinase, muscle; GR, glutathione reductase; GPx, glutathione peroxidase; SOD1, superoxide dismutase 1; CAT, catalase; HO-1, heme oxygenase-1; FPN1; ferroportin 1; BLVR, biliverdin reductase; FTH, ferritin heavy chain; FTL, ferritin light chain; PDM, phosphoglucomutase; GBE1, glycogen branching enzyme. Metabolite abbreviations: G-6-P, glucose 6-phosphate; G-1-P, glucose 1-phosphate; F-6-P, fructose 6-phosphate; F-1,6-BP, fructose 1,6-bisphosphate; F-2,6-BP, fructose 2,6-bisphosphate; GA-3-P, glyceraldehyde 3-phosphate; 1,3-PG, 1,3-phosphoglycerate; 3-PG, 3-phosphoglycerate; 2-PG, 2-phosphoglycerate; PEP, phosphoenolpyruvate; UDP-G, uracil-diphosphate glucose; 6-P-GL, 6-phosphogluconolactone; 6-PG, 6-phosphogluconate; R-5-P, ribulose 5-phosphate; 5-PRA, phosphoribosylamine; PRPP, 5-phosphoribosyl-α-1-pyrophosphate; THF, tetrahydrofolate; CH2-THF, 5,10-methylene-tetrahydrofolate; CHO-THF, 10-formyl- tetrahydrofolate; IMP, inosine monophosphate; AMP, adenosine monophosphate; GMP, guanosine monophosphate. Gly, glycine; Ala, alanine; Cys, cysteine; Glu, glutamate; Gln, glutamine; aKG, a-ketoglutarate; Y-Glu-Cys, gamma-glutamyl cysteine; Acetyl-CoA, acetyl-coenzyme A; NADPH, Nicotinamide adenine dinucleotide phosphate, reduced.

Environmental cues can disrupt the cellular functions, including metabolic changes. Transient NRF2 activation as a stress response mediates the cellular programs trying to maintain the homeostasis if the damage caused by the toxic stimuli are reparable. NRF2 affects multiple aspects of metabolism and mitochondrial function, from nutrients uptake, anabolic metabolism, macromolecular biosynthesis to energy metabolism that support cell growth and proliferation [76]. A plethora of evidence supports a key role for NRF2 in the cancer metabolism reprogramming [23]. Most of the evidence came from the Chromatin Immunoprecipitation Sequencing (ChIP-Seq) analysis of NRF2 target genes in cell lines [35,49,50] or in mouse tissues [77] where NRF2 is either activated or deleted to compare with the wild type cells. Those studies identified numerous NRF2 target genes that regulate glycolysis, pentose phosphate pathway (PPP), fatty acid metabolism, glutamine metabolism, and glutathione metabolism (Figure 3). The effects of NRF2 on the metabolism can be non-autonomous. The increased NRF2 activation in the cancer leads to secreted metabolites which can affect the tumor microenvironment [78]. In the brain, NRF2 expression is higher in the astrocytes and microglia than neurons [79]. Crosstalk between astrocytes and neurons couples intermediate metabolism with redox homeostasis and neighboring astrocytes provides neurons with the GSH precursors including glycine, glutamate/glutamine and cysteine, as well as other metabolites to support neurons functioning [80].

### 4.1. NRF2 Upregulates Aerobic Glycolysis and Glycogen Synthesis, but Inhibits Gluconeogenesis

Constitutive activation of NRF2 has been observed in many human cancers that have poor prognoses and NRF2 contributes to aerobic glycolysis to produce anabolic precursors for the building blocks of tumor growth with much less efficient energy production, a state known as the Warburg effect [23]. NRF2 induces expression of glucose transporter GLUT1 that allows increased glucose import into glycolytic flux [77]. Among the glycolysis pathway, NRF2 induces the expression of several key glycolytic enzymes, including hexokinase 1 and 2 (HK1/2), glucose phosphate isomerase 1 (GPI1), 6-phosphofructo-2-kinase (PFK2), PFK4, fructose-bisphosphate aldolase A (ALDA), enolase 1 (ENO1), ENO4, pyruvate kinase muscle isoform 2 (PKM2) to increase glycolytic flow and maintain pool sizes of glycolytic intermediates for anabolic reactions [77]. Pyruvate kinase (PK) catalyzes the final step in glycolysis, converting phosphoenolpyruvate (PEP) to pyruvate while phosphorylating ADP to ATP. PKM2 is highly expressed in cancer cells that coordinates high energy requirements with high anabolic activities to support cancer cell proliferation [81]. Interestingly, pyruvate kinase liver and red blood cell (PKLR) expression is inhibited in liver-specific *Keap1*-null mice [82]. A decrease in PK activity would favor buildup of glycolytic intermediates and their channeling into the synthesis of amino acids, nucleic acids, and phospholipids.

Diabetic db/db mice crossed with *Keap1* gene hypomorphic knockdown (*Keap1*^flox/−^) mice has revealed that genetic activation of NRF2 inhibits gluconeogenesis by suppressing glucose-6-phosphatase (G6PC), fructose-1,6-bisphosphatase 1 (FBP1), phosphoenolpyruvate carboxykinase (PCK1), peroxisome proliferator-activated receptor g coactivator 1-a (PGC1a), and nuclear receptor subfamily 4, group A, member 2 (NR4A2) in the liver, thereby preventing onset of Diabetes Mellitus [83]. Consistent with this study, liver-specific constitutive NRF2 activation also reduces gluconeogenesis-related gene PCK1 and pyruvate dehydrogenase kinase (PDK1) [75].

Glucose-6-phosphate, the first product of glycolysis, can also be converted to glucose-1-phosphate by phosphoglucomutase (PGM) for glycogen synthesis and NRF2 activates the expression of PGM5, 1,4-alpha-glucan branching enzyme 1 (GBE1), phosphorylase kinase regulatory subunit alpha 1 (PHKA1), and glucosidase alpha, acid (GAA) involved in glycogen metabolism [35,77,84]. The liver and skeletal muscle are the two major organs responsible for glycogen synthesis and storage and NRF2 differentially regulates glycogen metabolism in muscle [84]. Constitutive NRF2 activation in mouse liver increases glycogen accumulation that contributes to the maintenance of blood glucose levels during fasting [75,84], while skeletal muscle-specific *Keap1*-knockout mice show decreased glycogen content in skeletal muscle, accompanied with the improved glucose tolerance [84].

### 4.2. NRF2 Induces Expression of Genes Encoding Enzymes Involved in the Pentose Phosphate Pathway (PPP)

The first glycolytic intermediate, glucose-6-phosphate, can be diverted to PPP by glucose-6-phosphate dehydrogenase (G6PD) and phosphogluconate dehydrogenase (PGD), the two key enzymes in the oxidative phase of the PPP. Both G6PD and PGD are controlled by NRF2 [35,77]. G6PD and PGD also mediate the generation of NADPH as reducing equivalents, which is required for the biosynthesis of lipids and nucleotides and contributes to the increased cell proliferation in tumors in which NRF2 is upregulated [23,35]. Besides G6PD and PGD, malic enzyme (ME1) and isocitrate dehydrogenase (IDH1) also catalyze the production of NADPH [35,77]. In addition, NRF2 induces expression of genes encoding for transketolase (TKT) and transaldolase 1 (TALDO1), two enzyme for nonoxidative phase of the PPP [35]. NRF2 can directly activate G6PD, PGD, TKT, and TALDO1 by binding to the well-conserved AREs in their promoters, thereby directing carbon flux towards the PPP [35]. However, it was reported that NRF2 can also indirectly regulate G6PD, PGD, and TKT by downregulating the expression of miR-1 and miR-206 [85].

### 4.3. NRF2 Activates the de Novo Purine Biosynthesis Pathway

Two major products of the PPP, ribose-5-phosphate and erythrose-4-phosphate, are precursors for the biosynthesis of nucleotides and aromatic amino acids, respectively. These metabolites in the nucleotide and amino acid biosynthetic pathways provide continuous flux through the PPP. Ribose-5-phosphate is converted to 5-phospho-ribosyl-a-1-pyrophosphate (PRPP), which then is catalyzed by phosphoribosyl pyrophosphate amidotransferase (PPAT) to generate phosphoribosylamine (5PRA), a rate-limiting step in the de novo purine biosynthetic pathway. Methylenetetrahydrofolate dehydrogenase 2 (MTHFD2), a bifunctional enzyme with methylenetetrahydrofolate dehydrogenase and methenyltetrahydrofolate cyclohydrolase activities, provides one-carbon units for purine biosynthesis. Both PPAT and MTHFD2 are direct NRF2 target genes [23,35]. A tracer study using (U-^13^C_6_) glucose and dialyzed FBS revealed that the biosynthesis of purine metabolites, such as inosine monophosphate (IMP), AMP, and ATP, is increased in *Keap1*-knockout MEFs and decreased in their *Nfe2l2*-knockdown counterparts [35].

### 4.4. NRF2 Promotes Amino Acid Metabolism

In nonproliferating cells, the glycolysis end product pyruvate enters the tricarboxylic acid (TCA) cycle for maximal ATP production via oxidative phosphorylation. In proliferating cells, besides providing ATP, the TCA cycle serves as an important source of biosynthetic precursors. As a result, TCA cycle intermediates must be replenished via a process called anaplerosis. Glutamine, the most abundant amino acid in human plasma and the obligatory nitrogen donor for the biosynthesis of nucleotides and nonessential amino acids, is a major contributor together with glucose to anaplerotic flux [86]. Glutamine transporter solute carrier family 1 member 5 (SLC1A5) mediates glutamine uptake and it is transcriptionally activated by NRF2 [87]. In addition, two key glutaminolysis enzymes glutaminase (GLS2) and glutamic pyruvate transaminase 2 (GPT2) are activated by NRF2 and they control the production of glutamate, aspartate, alanine, and α-ketoglutarate, which are needed for nucleotides and nonessential amino acids synthesis in cancer cells [77]. Upon conversion to α-ketoglutarate, glutamine is an energy and anaplerotic carbon source that replenishes TCA intermediates. A glutamine tracing study has shown that the carbon flux from glutamine is directed towards GSH synthesis and the TCA cycle in NRF2 activated A549 cells [35].

NRF2 also regulates de novo serine biosynthesis in cooperation with ATF4, leading to synthesis of serine-derived glycine and cysteine via the methionine cycle and one carbon metabolism [23,35,88]. Glutamate, cysteine and glycine are used for glutathione synthesis, catalyzed by GCLC, GCLM, and GSS, which are all NRF2 targets [23]. Glutamate is also an obligate exchange molecule for the NRF2-regulated glutamate–cystine antiporter (xCT) encoded by solute carrier family 7 member 11 (SLC7A11), which controls the intracellular availability of cysteine [77,89]. Activation of NRF2 in cancer cells imposes a glutamine addition that is caused by an increased dependency on exogenous glutamine through increased consumption of glutamate for glutathione synthesis and glutamate secretion by xCT antiporter system, thus limiting glutamate availability for the TCA cycle and other biosynthesis pathway [89].

### 4.5. NRF2 Regulates Lipid Metabolism

NRF2 regulates triglycerides/phospholipids degradation and synthesis, lipid transport as well as fatty acid oxidation. The lipid metabolism mediated by NRF2 is cell type- and context-dependent [50,77,82]. *Nfe2l2*-null C57BL/6 mice exhibited a tendency towards higher hepatic triglycerides compared to wild-type mice on a high-fat diet (HFD) feeding and *Nfe2l2* deletion dysregulates hepatic expression of gene involved in fatty acid β-oxidation [90]. However, *Nfe2l2*-null mice on C57BL6;129SV mix background in mice showed decreased adipose tissue mass, formation of small adipocytes, and protected against weight gain and obesity induced by HFD [91]. Liver specific *Keap1*-null (Alb-Cre:*Keap1*^flox/−^) mice exhibit reduced liver free fatty acids and triglycerides [82]. *Keap1*-knockdown in 3T3-L1 cells enhances adipocyte differentiation [91]. Esophagus of *Keap1*-null mice shows elevated phospholipids and long chain free fatty acid (FFA) [77]. In addition, constitutive NRF2 activation in hepatocytes results in liver triglyceride accumulation [75]. CHIP-Seq analysis revealed that genes encoding for several key enzymes in lipid metabolism, including elongation of very long chain fatty acids protein 7 (ELOVL7), acyl-CoA synthetase short-chain family member 2 (ACSS2), acyl-CoA thioesterase 7 (ACOT7), fatty acid desaturase 1 (FADS1), acyl-Coenzyme A dehydrogenase family member 10 (ACAD10), and acyl-Coenzyme A dehydrogenase family member 12 (ACAD12) are activated by NRF2 [77]. In addition, NRF2 also activates CD36, the lipid uptake transporter/receptor in different cells to regulate lipid metabolism [92].

### 4.6. NRF2 Regulates Heme and Iron Metabolism

Heme is a coordination complex consisting of an iron ion coordinated to a porphyrin acting at the center of many mitochondria complexes and cytochrome P-450 enzymes, nitric oxide signaling nitric oxide synthases, oxygen storage protein myoglobin, and oxygen carrier protein hemoglobin. Because iron can promote the formation of damaging oxygen radicals, synthesis and destruction of iron-bound heme are carefully regulated. Ferrochelatase (FECH), a key enzyme that carries out the last step of heme biosynthesis by inserting ferrous iron into protoporphyrin to generate the final heme cofactor, is a direct NRF2 target gene [50]. NRF2 also alters iron homeostasis by increasing iron storage and its flux in and out of the cell. Intracellular iron storage protein ferritin, including ferritin heavy chain (FTH) and ferritin light chain (FTL), sequesters excess free iron in a protein cage that limits iron’s redox switching. Ferroportin 1 (FPN1), the only known mammalian exporter of iron from the cytosol to the extracellular milieu, regulates iron reutilization. NRF2 induces expression of genes encoding both FTL, FTH, and FPN1 [50,93]. Cytosolic heme is catabolized into ferrous iron and biliverdin by HO-1, an NRF2 target gene [54]. Biliverdin is further metabolized to bilirubin, which subsequently serves as an antioxidant free radical scavenger and can be glucuronidated for excretion, by either biliverdin reductase A (BLVRA) or biliverdin reductase B (BLVRB). Expression of both BLVRs depends on NRF2 [94].

## 5. NRF2 Regulates Unfolded Protein Response (UPR) and Proteostasis

Proteostasis refers to the homeostatic control of synthesis, folding, trafficking, and degradation of proteome. Aberrant aggregation of misfolded or denatured proteins has been observed in many diseases, such as cardiovascular disease, metabolic syndrome, cancer, and neurodegenerative disease [95]. Accumulation of misfolded proteins that lead to ER stress triggers the UPR via activation of three signaling arms coordinated by IRE1-XBP1, PERK-eIF2a-ATF4, and ATF6 [78]. UPR is a highly conserved pathway that has evolved to respond to protein misfolding in the ER. Misfolded protein accumulation and aggregation induce excessive production of ROS from mitochondria, ER, and other sources, which can activate NRF2 [95,96]. In addition, stress-induced PERK phosphorylates NRF2, resulting in dissociation of NRF2/KEAP1 complexes and NRF2 activation [27]. NRF2 is a hub that compiles emergency signals derived from misfolded protein accumulation to facilitate a coordinated transcriptional response. The NRF2 homolog in C. elegans SKN-1 induces several components of the UPR target genes, including XBP1 and ATF6, which induce a UPR program for maintaining ER integrity and protein homeostasis [97], although the induction of its UPR target genes may be through NRF1 [98]. NRF2 activates the transcription of ATF4, which has been linked to amino acid metabolism and resistance to oxidative stress [88,99]. NRF2 and ATF4 form a heterocomplex to induce the target genes expression to survive proteotoxic stress [54]. NRF2 activation in mice liver induces the expression of genes involved in the UPR [75]. In addition, NRF2 activation in zebrafish reduces ER stress induced by a mutation in the phosphomannomutase 2 gene, which results in mild defects in the initial steps of *N*-glycosylation [100].

Damaged, misfolded, oxidized, or short-lived proteins are degraded by 26S proteasome, which consists of a 20S core and a 19S regulatory subunit. NRF2 regulates the basal and inducible expression of genes of multiple subunits of the 20S proteasome, including PSMA1, PSMA4, PSMB3, PSMB5, and PSMB6, as well as 19S proteasome subunits PSMC1, PSMC3, and PSMD14 [101]. There are several ARE-like motifs in PSMB5 [101]. Interestingly, NRF2 seems to play little role in the expression of immunoproteasome [102]. Regulation of the proteasome by NRF2 is conserved between mice and humans [101,103,104]. In addition, NRF2 binds to the promoter of gene encoding proteasome maturation protein (POMP), which mediates proteasome assembly, and induces its expression [105,106]. Elevated NRF2 activation in cancers is associated with increased proteasome activity and resistance to the proteasome inhibitor bortezomib [107]. In summary, NRF2 increases proteasome activity, together with anti-oxidant expression, and contributes to the stress adaptation.

## 6. NRF2 Regulates Autophagy

Autophagy is a protein and organelle quality control mechanism that orderly degrades and recycles cellular components, including protein aggregates and old or damaged organelles. Beside ubiquitin proteasome system, oxidative, proteotoxic, and metabolic stresses increase autophagy and help to restore homeostasis [108]. Autophagy requires the coordinated participation of a set of proteins that participate in the formation of autophagosomes and autolysosomes, as well as cargo-selective proteins that recognize specific cargos and direct them to degradation. NRF2 induces the expression of autophagy genes encoding SQSTM1/p62, calcium-binding and coiled-coil domain-containing protein 2 (CALCOCO2/NDP52), unc-51-like kinase 1 (ULK1), autophagy protein 5 (ATG5), and gamma-aminobutyric acid receptor-associated protein-like 1 (GABARAPL1) and enhances autophagy [109]. In the NRF2 activated context, autophagy-targeted therapy could be ineffective. Interestingly, insufficient autophagy leads to accumulation of oxidized proteins or organelles that can lead to NRF2 activation. More importantly, autophagy deficiency leads to accumulation of p62, a multifunctional cargo receptor that can sequester KEAP1 and stabilize NRF2, resulting in NRF2 activation [10]. Thus, p62 and NRF2 create a positive feedback loop to regulate a plethora of cellular functions [9,110].

## 7. NRF2 Regulates Mitochondrial Physiology and Biogenesis

The mitochondria provide the cell with energy from oxidative phosphorylation, which is intimately linked to the production of ROS. Imbalance in the generation of ROS is a common feature in several diseases, including neurodegeneration disorders, metabolic disorders, cardiovascular disease, and cancer [80]. NRF2 affects multiple aspects of intermediary metabolism, antioxidant response, and mitochondrial function through the regulation of some key metabolic genes or through crosstalk with other transcription factors. NRF2 activation enhances glycolytic flux, PPP, amino acid metabolism, and glutaminolysis, resulting in an increased entry of their substrates and reducing equivalents into the TCA cycle and the mitochondrial respiratory chain. Cells with genetic activation of NRF2 have higher oxygen consumption rates, higher basal mitochondrial membrane potential (ΔΨm), and higher basal ATP levels, while the *Nfe2l2*-deficient cells have opposite phenotypes [111]. Mitochondrial fatty acid oxidation is impaired in *Nfe2l2*-null cells and accelerated when NRF2 is constitutively active [112]. High oxidative phosphorylation increases mitochondrial electron leak and thus increases ROS levels. However, NRF2 activation directly upregulates the uncoupling protein 3 (UCP3) that uncouples respiration from oxidative phosphorylation to allow energy to be released as heat, thus decreasing superoxide formation [113]. NRF2 activation also maintains the integrity of mitochondrial DNA (mtDNA), which controls cell death and inflammation [114].

NRF2 stimulates the mitochondrial biogenesis program through activation of nuclear respiratory factor-1 (NRF-1), which transcribes the key mitochondrial biogenesis factors transcription factor A, mitochondrial (TFAM) and transcription factor B2, mitochondrial (TFBM2) [115]. Moderate physical exercise induces NRF2-dependent mitochondrial biogenesis in muscles [116] and in the striatum in the 6-OHDA-induced model of parkinsonism [117]. In addition, NRF2 directly controls expression of transcriptional co-activator peroxisome proliferator activated receptor gamma co-activator 1 alpha (PGC-1a), a master regulator of mitochondrial function and biogenesis [118]. NRF2 and PGC-1a together regulate almost all aspects of mitochondria functions [118].

## 8. NRF2 Regulates Inflammation and Immunity

**Figure 4:**
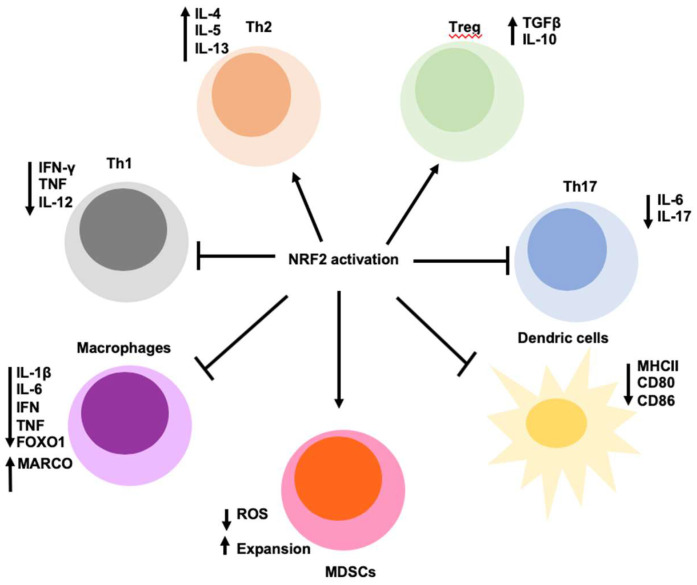
NRF2 regulates inflammation and immunity. The activation of NRF2 may alter the differentiation, expansion, and survival of the immune cells as well as the cytokine release. NRF2 activation shifts Th1/Th2 balance in disease models by impairing Th1-driven response and skews them towards Th2 differentiation. NRF2-mediated antioxidant defense in Treg cells leads to their expansion and survival. *Nfe2l2*-deficiency leads to elevated oxidative damage that exacerbates the differentiation of Th17 cells. *Nfe2l2* deletion in dendritic cells augments expression of MHC class II and the cells surface expression of co-stimulatory molecules CD86 and CD80 to influence the behavior of Th cells. Increased NRF2 expression in leukocytes such as macrophages inhibits the expression of pro-inflammatory genes through down-regulation of the NF-κB pathway. NRF2 activation in MDSCs regulates metabolism that leads to expansion of suppressive MDSCs. However, *Nfe2l2*-deletion in MDSCs have higher levels of intracellular ROS that suppress CTLs proliferation and induces T-cell anergy.

NRF2 is ubiquitously expressed although at different levels in different cell types [69]. In blood, monocytes, neutrophils, T cells, and B cells exhibit the high levels of NRF2 [119], suggesting an immunomodulatory effect of the immune system. In addition, in the brain, NRF2 transcripts are higher in microglia, part of the monocyte lineage than neurons and endothelia cells [79]. Inflammation is triggered when innate immune cells detect infection or tissue injury. Inflammation protects the host from infection, illness, or injury by eliminating impending injury or infection and initiates tissue repair. The ROS act as both a signaling molecule and a mediator of inflammation [23]. However, uncontrolled inflammation can lead to cell damage or cellular hyperplasia following ROS overproduction from inflammatory cells, resulting in chronic inflammation. Chronic inflammation underlies many chronic diseases including diabetes, metabolic syndrome, cardiovascular disease, cancer, autoimmune diseases, and respiratory disease [80]. Considering a major role of NRF2 activation in the modulation of redox metabolism that attenuates inflammation-associated ROS, NRF2 is anti-inflammation (Figure 4). *Nfe2l2*-deficient mice are hypersensitive to septic shock [120], display more severe lung inflammation induced by cigarette smoke [121], are highly susceptible in different liver inflammation models [122]. In addition, *Nfe2l2*-null mice tend to develop age-dependent autoimmune phenotypes in certain genetic background [123,124]. Genetic or pharmacological activation of NRF2 suppresses acute inflammatory liver injury [125] and neuroinflammation [126]. In addition, NRF2 blocks inflammation by directly inhibiting transcription of the proinflammatory cytokine genes or inhibiting the activity of inflammatory nuclear factor kappa B (NF-κB) signaling [23]. ChIP-seq analysis revealed that NRF2 directly binds to the promoter proximal regions of IL-6 and IL-1β genes to disrupt RNA polymerase II recruitment and blocks gene induction in macrophages [127]. NRF2 can also prevent IL-6 expression by ARE-dependent induction of ATF3 that suppresses IL-6 transcription [128,129]. However, inhibition of pro-inflammatory cytokines by NRF2 is cell type- and context- dependent. In hepatocytes [130] and in persistent polyclonal B cell lymphocytosis B cells [131], NRF2 activation directly induces the transcription of IL-6. Furthermore, NRF2 directly upregulates the expression of MARCO gene, encoding a scavenger receptor required for bacterial phagocytosis, thereby improving bacterial clearance [132].

NRF2 can either suppress or promote host immunity in a cell type- and disease context-dependent manner (Figure 4). Immunological surveillance is a monitoring process of the immune system to detect and destroy virally infected and neoplastically transformed cells in the body. Metabolic reprogramming mediated by NRF2 modulates immune cell functions. NRF2 activation decreases STING expression by destabilizing its mRNA, which leads to decreased type I IFN production, decreased antiviral cytosolic DNA sensing, and increased DNA virus infection in human cells, whereas the silencing of NRF2 decreases virus infectivity [133]. Marburg virus (MARV) hijacks KEAP1-NRF2 signaling by targeting KEAP1, thus activating NRF2, in order to decrease host immune responses [134]. However, NRF2 activation in tumor cells or virally infected cells induces IL-17D and thereby potentiating anti-tumor immunity and antiviral immunity against vaccina virus (VV) and mouse cytomegalovirus (MCMV) [135]. The basis for these different effects on antiviral immunity is not known and further study of mechanism of NRF2 antiviral immunity is warranted.

Anti-tumor immunity is determined by both the tumor microenvironment and tumor cells. Cancers arise through cancer immunoediting to eventually escape the immune system [136]. Anti-tumor immunity is mainly mediated by CD8^+^ cytotoxic T lymphocytes (CTLs), CD4^+^ Th1 helper cells, and natural killer (NK) cells [78,137]. Immune suppression in cancer is mainly mediated by regulatory T (Treg) cells and myeloid-derived suppressor cells (MDSCs), which comprise a heterogeneous group of immune cells from the myeloid lineage including tumor-associated macrophages, dendritic cells, and other immature myeloid cells. Constitutive NRF2 activation in MDSCs regulates activation of glycolysis and mitochondrial metabolism that leads to expansion of suppressive MDSCs and acquired tolerance against LPS-induced sepsis [138]. However, *Nfe2l2*-null MDSCs have higher levels of intracellular ROS that suppress CTLs proliferation and induces T-cell anergy, a state of loss of CTLs antigen recognition, resulting in the increased tumor metastasis in a xenograft model of lung cancer [139,140]. *Nfe2l2* deletion in the bone marrow-derived macrophages (BMDM) decreases the levels of GSH by downregulation of GCLM and xCT, which leads to limited the GSH availability to CTLs, thereby inhibiting antigen-induced CTLs proliferation and function [141]. Activated NRF2 in cancer cells can induce IL-17D expression that recruits NK cells, resulting in increased anti-tumor immunity and NK-dependent tumor regression [135]. NRF2 activation in T cells decreases T cell activation and differentiation. Systemic NRF2 activation by *Keap1*- deficiency and T cell-specific NRF2 activation decreases IFN-γ production by effector Th1 and CTLs in the scurfy mode [142]. NRF2 activation by NRF2 activators decreases IFN-γ production and increases IL-4, IL-5, and IL-13 production in CD4+ T cells and skews them towards Th2 differentiation [143,144]. NRF2 inhibition also alters the phenotype dendritic cells. NRF-deficient dendritic cells have impaired GSH levels, reduced phagocytic activity, augmented expression of MHC class II, and enhanced co-stimulatory receptor expression of CD86 and CD80, thereby enhancing T cells stimulatory capacity [145]. NRF2 activation inhibits the phosphorylation of Th17 transcription factor STAT3 and decreases Th17 differentiation [146], but increases the expansion and activity of Treg cells [147,148]. In human ovarian cancer, weak NRF2-associated antioxidant defense in Treg cells leads to apoptosis induced by high ROS in tumor microenvironment and the apoptotic Treg cells abolish spontaneous and PD-L1-blockade-mediated antitumor T cell immunity, but sustain and amplify antitumor immunity via the adenosine receptor pathway [149]. Activation and expansion of immune cells paradoxically depend not only on ROS, but also on the ability to limit ROS. Considering that many of the above results are derived from the use of whole body *Nfe2l2*-knockout mice, *Keap1*-knockout mice, or pharmacological NRF2 activators of questioned specificity, continued research into the function of NRF2 in specific immune cell subsets is highly warranted. Since NRF2 activation in cancer cells is pro-tumorigenic, it is likely that it attenuates anti-tumor immunity. It is premature to conclude a definite role of NRF2 activation in immune cells without considering the disease context.

## 9. Concluding Remarks and Perspectives

Although NRF2 is best known as an oxidant stress response transcription factor, it is also critical to the regulation of metabolism, inflammation, autophagy, proteostasis, mitochondrial physiology, and immune responses. NRF2-mediated target gene expression is determined by the activating stimulus, the cellular context, the availability of binding partners, interaction with other transcription factors, activators, and repressors, as well as the crosstalk with other signaling pathways. NRF2 lies at the center of a complex regulatory network and most of functions exerted by NRF2 are interconnected that contribute to the initiation and development of many diseases, particularly metabolism- or inflammation-associated diseases. Generally, NRF2 activation plays a protective role in physiological conditions, but it promotes cancer development after cancer is established. In addition, NRF2 is generally anti-inflammation, however, it exhibits opposite roles during different virus infections. Future studies need to address how the NRF2-dependent network changes from a physiological (beneficial) to a pathological (adversary) condition that can provide insights into mechanisms of disease pathogenesis. The key switch between the tumor suppressive role and tumor promoting roles of NRF2 could be due to the active NRF2 protein levels/doses and the duration of NRF2 activation in the crosstalk with specific factors and signaling pathways. In addition, due to the broad action spectrum, understanding more dynamic and integrated NRF2-mediated metabolism and immunity of different cell types in a disease context will facilitate discovery of molecular pathology and new treatment strategies.

## Abbreviations

NRF2	Nuclear factor erythroid 2-Related Factor 2
NFE2L2	Nuclear Factor, Erythroid 2 Like 2
KEAP1	Kelch-like-ECH-associated protein 1
βTrCP	β-Transducin repeat-Containing Protein

## References

[1] Jaramillo M.C., Zhang D.D. (2013). The emerging role of the Nrf2-Keap1 signaling pathway in cancer. Genes Dev..

[2] Taguchi K., Yamamoto M. (2017). The KEAP1-NRF2 System in Cancer. Front. Oncol..

[3] Canning P., Sorrell F.J., Bullock A.N. (2015). Structural basis of Keap1 interactions with Nrf2. Free Radic. Biol. Med..

[4] McMahon M., Thomas N., Itoh K., Yamamoto M., Hayes J.D. (2004). Redox-regulated turnover of Nrf2 is determined by at least two separate protein domains, the redox-sensitive Neh2 degron and the redox-insensitive Neh6 degron. J. Biol. Chem..

[5] Wang H., Liu K., Geng M., Gao P., Wu X., Hai Y., Li Y., Li Y., Luo L., Hayes J.D. (2013). RXRalpha inhibits the NRF2-ARE signaling pathway through a direct interaction with the Neh7 domain of NRF2. Cancer Res..

[6] McMahon M., Lamont D.J., Beattie K.A., Hayes J.D. (2010). Keap1 perceives stress via three sensors for the endogenous signaling molecules nitric oxide, zinc, and alkenals. Proc. Natl. Acad. Sci. USA.

[7] Baird L., Lleres D., Swift S., Dinkova-Kostova A.T. (2013). Regulatory flexibility in the Nrf2-mediated stress response is conferred by conformational cycling of the Keap1-Nrf2 protein complex. Proc. Natl. Acad. Sci. USA.

[8] Itoh K., Wakabayashi N., Katoh Y., Ishii T., O’Connor T., Yamamoto M. (2003). Keap1 regulates both cytoplasmic-nuclear shuttling and degradation of Nrf2 in response to electrophiles. Genes Cells.

[9] Taniguchi K., Yamachika S., He F., Karin M. (2016). p62/SQSTM1-Dr. Jekyll and Mr. Hyde that prevents oxidative stress but promotes liver cancer. FEBS Lett..

[10] Komatsu M., Kurokawa H., Waguri S., Taguchi K., Kobayashi A., Ichimura Y., Sou Y.S., Ueno I., Sakamoto A., Tong K.I. (2010). The selective autophagy substrate p62 activates the stress responsive transcription factor Nrf2 through inactivation of Keap1. Nat. Cell Biol..

[11] Umemura A., He F., Taniguchi K., Nakagawa H., Yamachika S., Font-Burgada J., Zhong Z., Subramaniam S., Raghunandan S., Duran A. (2016). p62, Upregulated during Preneoplasia, Induces Hepatocellular Carcinogenesis by Maintaining Survival of Stressed HCC-Initiating Cells. Cancer Cell.

[12] Hast B.E., Goldfarb D., Mulvaney K.M., Hast M.A., Siesser P.F., Yan F., Hayes D.N., Major M.B. (2013). Proteomic analysis of ubiquitin ligase KEAP1 reveals associated proteins that inhibit NRF2 ubiquitination. Cancer Res..

[13] Camp N.D., James R.G., Dawson D.W., Yan F., Davison J.M., Houck S.A., Tang X., Zheng N., Major M.B., Moon R.T. (2012). Wilms tumor gene on X chromosome (WTX) inhibits degradation of NRF2 protein through competitive binding to KEAP1 protein. J. Biol. Chem..

[14] Ma J., Cai H., Wu T., Sobhian B., Huo Y., Alcivar A., Mehta M., Cheung K.L., Ganesan S., Kong A.N. (2012). PALB2 interacts with KEAP1 to promote NRF2 nuclear accumulation and function. Mol. Cell. Biol..

[15] Chen W., Sun Z., Wang X.J., Jiang T., Huang Z., Fang D., Zhang D.D. (2009). Direct interaction between Nrf2 and p21(Cip1/WAF1) upregulates the Nrf2-mediated antioxidant response. Mol. Cell.

[16] Gorrini C., Baniasadi P.S., Harris I.S., Silvester J., Inoue S., Snow B., Joshi P.A., Wakeham A., Molyneux S.D., Martin B. (2013). BRCA1 interacts with Nrf2 to regulate antioxidant signaling and cell survival. J. Exp. Med..

[17] Ganner A., Pfeiffer Z.C., Wingendorf L., Kreis S., Klein M., Walz G., Neumann-Haefelin E. (2020). The acetyltransferase p300 regulates NRF2 stability and localization. Biochem. Biophys. Res. Commun..

[18] Chowdhry S., Zhang Y., McMahon M., Sutherland C., Cuadrado A., Hayes J.D. (2013). Nrf2 is controlled by two distinct beta-TrCP recognition motifs in its Neh6 domain, one of which can be modulated by GSK-3 activity. Oncogene.

[19] Hayes J.D., Chowdhry S., Dinkova-Kostova A.T., Sutherland C. (2015). Dual regulation of transcription factor Nrf2 by Keap1 and by the combined actions of beta-TrCP and GSK-3. Biochem. Soc. Trans..

[20] Lo J.Y., Spatola B.N., Curran S.P. (2017). WDR23 regulates NRF2 independently of KEAP1. PLoS Genet..

[21] Wu T., Zhao F., Gao B., Tan C., Yagishita N., Nakajima T., Wong P.K., Chapman E., Fang D., Zhang D.D. (2014). Hrd1 suppresses Nrf2-mediated cellular protection during liver cirrhosis. Genes Dev..

[22] Kang H.J., Hong Y.B., Kim H.J., Bae I. (2010). CR6-interacting factor 1 (CRIF1) regulates NF-E2-related factor 2 (NRF2) protein stability by proteasome-mediated degradation. J. Biol. Chem..

[23] He F., Antonucci L., Karin M. (2020). NRF2 as a regulator of cell metabolism and inflammation in cancer. Carcinogenesis.

[24] Yuan X., Xu C., Pan Z., Keum Y.S., Kim J.H., Shen G., Yu S., Oo K.T., Ma J., Kong A.N. (2006). Butylated hydroxyanisole regulates ARE-mediated gene expression via Nrf2 coupled with ERK and JNK signaling pathway in HepG2 cells. Mol. Carcinog..

[25] Huang H.C., Nguyen T., Pickett C.B. (2002). Phosphorylation of Nrf2 at Ser-40 by protein kinase C regulates antioxidant response element-mediated transcription. J. Biol. Chem..

[26] Apopa P.L., He X., Ma Q. (2008). Phosphorylation of Nrf2 in the transcription activation domain by casein kinase 2 (CK2) is critical for the nuclear translocation and transcription activation function of Nrf2 in IMR-32 neuroblastoma cells. J. Biochem. Mol. Toxicol..

[27] Cullinan S.B., Zhang D., Hannink M., Arvisais E., Kaufman R.J., Diehl J.A. (2003). Nrf2 is a direct PERK substrate and effector of PERK-dependent cell survival. Mol. Cell. Biol..

[28] Sanghvi V.R., Leibold J., Mina M., Mohan P., Berishaj M., Li Z., Miele M.M., Lailler N., Zhao C., De Stanchina E. (2019). The Oncogenic Action of NRF2 Depends on De-glycation by Fructosamine-3-Kinase. Cell.

[29] Kwak M.K., Itoh K., Yamamoto M., Kensler T.W. (2002). Enhanced expression of the transcription factor Nrf2 by cancer chemopreventive agents: Role of antioxidant response element-like sequences in the nrf2 promoter. Mol. Cell. Biol..

[30] Miao W., Hu L., Scrivens P.J., Batist G. (2005). Transcriptional regulation of NF-E2 p45-related factor (NRF2) expression by the aryl hydrocarbon receptor-xenobiotic response element signaling pathway: Direct cross-talk between phase I and II drug-metabolizing enzymes. J. Biol. Chem..

[31] Nair S., Doh S.T., Chan J.Y., Kong A.N., Cai L. (2008). Regulatory potential for concerted modulation of Nrf2- and Nfkb1-mediated gene expression in inflammation and carcinogenesis. Br. J. Cancer.

[32] Rushworth S.A., Zaitseva L., Murray M.Y., Shah N.M., Bowles K.M., MacEwan D.J. (2012). The high Nrf2 expression in human acute myeloid leukemia is driven by NF-kappaB and underlies its chemo-resistance. Blood.

[33] Rushworth S.A., MacEwan D.J., O’Connell M.A. (2008). Lipopolysaccharide-induced expression of NAD(P)H:quinone oxidoreductase 1 and heme oxygenase-1 protects against excessive inflammatory responses in human monocytes. J. Immunol..

[34] DeNicola G.M., Karreth F.A., Humpton T.J., Gopinathan A., Wei C., Frese K., Mangal D., Yu K.H., Yeo C.J., Calhoun E.S. (2011). Oncogene-induced Nrf2 transcription promotes ROS detoxification and tumorigenesis. Nature.

[35] Mitsuishi Y., Taguchi K., Kawatani Y., Shibata T., Nukiwa T., Aburatani H., Yamamoto M., Motohashi H. (2012). Nrf2 redirects glucose and glutamine into anabolic pathways in metabolic reprogramming. Cancer Cell.

[36] Wakabayashi N., Skoko J.J., Chartoumpekis D.V., Kimura S., Slocum S.L., Noda K., Palliyaguru D.L., Fujimuro M., Boley P.A., Tanaka Y. (2014). Notch-Nrf2 axis: Regulation of Nrf2 gene expression and cytoprotection by notch signaling. Mol. Cell. Biol..

[37] Yu S., Khor T.O., Cheung K.L., Li W., Wu T.Y., Huang Y., Foster B.A., Kan Y.W., Kong A.N. (2010). Nrf2 expression is regulated by epigenetic mechanisms in prostate cancer of TRAMP mice. PLoS ONE.

[38] Suzuki T., Shibata T., Takaya K., Shiraishi K., Kohno T., Kunitoh H., Tsuta K., Furuta K., Goto K., Hosoda F. (2013). Regulatory nexus of synthesis and degradation deciphers cellular Nrf2 expression levels. Mol. Cell. Biol..

[39] O’Brien J., Hayder H., Zayed Y., Peng C. (2018). Overview of MicroRNA Biogenesis, Mechanisms of Actions, and Circulation. Front. Endocrinol. (Lausanne).

[40] Sangokoya C., Telen M.J., Chi J.T. (2010). microRNA miR-144 modulates oxidative stress tolerance and associates with anemia severity in sickle cell disease. Blood.

[41] Yang M., Yao Y., Eades G., Zhang Y., Zhou Q. (2011). MiR-28 regulates Nrf2 expression through a Keap1-independent mechanism. Breast Cancer Res. Treat..

[42] Li N., Muthusamy S., Liang R., Sarojini H., Wang E. (2011). Increased expression of miR-34a and miR-93 in rat liver during aging, and their impact on the expression of Mgst1 and Sirt1. Mech. Ageing Dev..

[43] Singh B., Ronghe A.M., Chatterjee A., Bhat N.K., Bhat H.K. (2013). MicroRNA-93 regulates NRF2 expression and is associated with breast carcinogenesis. Carcinogenesis.

[44] Wang B., Teng Y., Liu Q. (2016). MicroRNA-153 Regulates NRF2 Expression and is Associated with Breast Carcinogenesis. Clin. Lab..

[45] Narasimhan M., Patel D., Vedpathak D., Rathinam M., Henderson G., Mahimainathan L. (2012). Identification of novel microRNAs in post-transcriptional control of Nrf2 expression and redox homeostasis in neuronal, SH-SY5Y cells. PLoS ONE.

[46] Hentze M.W., Castello A., Schwarzl T., Preiss T. (2018). A brave new world of RNA-binding proteins. Nat. Rev. Mol. Cell Biol..

[47] Poganik J.R., Long M.J.C., Disare M.T., Liu X., Chang S.H., Hla T., Aye Y. (2019). Post-transcriptional regulation of Nrf2-mRNA by the mRNA-binding proteins HuR and AUF1. FASEB J..

[48] Goldstein L.D., Lee J., Gnad F., Klijn C., Schaub A., Reeder J., Daemen A., Bakalarski C.E., Holcomb T., Shames D.S. (2016). Recurrent Loss of NFE2L2 Exon 2 Is a Mechanism for Nrf2 Pathway Activation in Human Cancers. Cell Rep..

[49] Malhotra D., Portales-Casamar E., Singh A., Srivastava S., Arenillas D., Happel C., Shyr C., Wakabayashi N., Kensler T.W., Wasserman W.W. (2010). Global mapping of binding sites for Nrf2 identifies novel targets in cell survival response through ChIP-Seq profiling and network analysis. Nucleic Acids Res..

[50] Chorley B.N., Campbell M.R., Wang X., Karaca M., Sambandan D., Bangura F., Xue P., Pi J., Kleeberger S.R., Bell D.A. (2012). Identification of novel NRF2-regulated genes by ChIP-Seq: Influence on retinoid X receptor alpha. Nucleic Acids Res..

[51] Hirotsu Y., Katsuoka F., Funayama R., Nagashima T., Nishida Y., Nakayama K., Engel J.D., Yamamoto M. (2012). Nrf2-MafG heterodimers contribute globally to antioxidant and metabolic networks. Nucleic Acids Res..

[52] Dhakshinamoorthy S., Jain A.K., Bloom D.A., Jaiswal A.K. (2005). Bach1 competes with Nrf2 leading to negative regulation of the antioxidant response element (ARE)-mediated NAD(P)H:quinone oxidoreductase 1 gene expression and induction in response to antioxidants. J. Biol. Chem..

[53] Reichard J.F., Motz G.T., Puga A. (2007). Heme oxygenase-1 induction by NRF2 requires inactivation of the transcriptional repressor BACH1. Nucleic Acids Res..

[54] He C.H., Gong P., Hu B., Stewart D., Choi M.E., Choi A.M., Alam J. (2001). Identification of activating transcription factor 4 (ATF4) as an Nrf2-interacting protein. Implication for heme oxygenase-1 gene regulation. J. Biol. Chem..

[55] Venugopal R., Jaiswal A.K. (1996). Nrf1 and Nrf2 positively and c-Fos and Fra1 negatively regulate the human antioxidant response element-mediated expression of NAD(P)H:quinone oxidoreductase1 gene. Proc. Natl. Acad. Sci. USA.

[56] Venugopal R., Jaiswal A.K. (1998). Nrf2 and Nrf1 in association with Jun proteins regulate antioxidant response element-mediated expression and coordinated induction of genes encoding detoxifying enzymes. Oncogene.

[57] Katoh Y., Itoh K., Yoshida E., Miyagishi M., Fukamizu A., Yamamoto M. (2001). Two domains of Nrf2 cooperatively bind CBP, a CREB binding protein, and synergistically activate transcription. Genes Cells.

[58] Ogryzko V.V., Schiltz R.L., Russanova V., Howard B.H., Nakatani Y. (1996). The transcriptional coactivators p300 and CBP are histone acetyltransferases. Cell.

[59] Brown S.L., Sekhar K.R., Rachakonda G., Sasi S., Freeman M.L. (2008). Activating transcription factor 3 is a novel repressor of the nuclear factor erythroid-derived 2-related factor 2 (Nrf2)-regulated stress pathway. Cancer Res..

[60] Shan Y., Akram A., Amatullah H., Zhou D.Y., Gali P.L., Maron-Gutierrez T., Gonzalez-Lopez A., Zhou L., Rocco P.R., Hwang D. (2015). ATF3 protects pulmonary resident cells from acute and ventilator-induced lung injury by preventing Nrf2 degradation. Antioxid. Redox Signal..

[61] Ki S.H., Cho I.J., Choi D.W., Kim S.G. (2005). Glucocorticoid receptor (GR)-associated SMRT binding to C/EBPbeta TAD and Nrf2 Neh4/5: Role of SMRT recruited to GR in GSTA2 gene repression. Mol. Cell. Biol..

[62] Zhang J., Ohta T., Maruyama A., Hosoya T., Nishikawa K., Maher J.M., Shibahara S., Itoh K., Yamamoto M. (2006). BRG1 interacts with Nrf2 to selectively mediate HO-1 induction in response to oxidative stress. Mol. Cell. Biol..

[63] Nioi P., Nguyen T., Sherratt P.J., Pickett C.B. (2005). The carboxy-terminal Neh3 domain of Nrf2 is required for transcriptional activation. Mol. Cell. Biol..

[64] Kim J.H., Yu S., Chen J.D., Kong A.N. (2013). The nuclear cofactor RAC3/AIB1/SRC-3 enhances Nrf2 signaling by interacting with transactivation domains. Oncogene.

[65] Pan H., Guan D., Liu X., Li J., Wang L., Wu J., Zhou J., Zhang W., Ren R., Zhang W. (2016). SIRT6 safeguards human mesenchymal stem cells from oxidative stress by coactivating NRF2. Cell Res..

[66] Sekine H., Okazaki K., Ota N., Shima H., Katoh Y., Suzuki N., Igarashi K., Ito M., Motohashi H., Yamamoto M. (2016). The Mediator Subunit MED16 Transduces NRF2-Activating Signals into Antioxidant Gene Expression. Mol. Cell. Biol..

[67] Ikeda Y., Sugawara A., Taniyama Y., Uruno A., Igarashi K., Arima S., Ito S., Takeuchi K. (2000). Suppression of rat thromboxane synthase gene transcription by peroxisome proliferator-activated receptor gamma in macrophages via an interaction with NRF2. J. Biol. Chem..

[68] Ansell P.J., Lo S.C., Newton L.G., Espinosa-Nicholas C., Zhang D.D., Liu J.H., Hannink M., Lubahn D.B. (2005). Repression of cancer protective genes by 17beta-estradiol: Ligand-dependent interaction between human Nrf2 and estrogen receptor alpha. Mol. Cell. Endocrinol..

[69] Moi P., Chan K., Asunis I., Cao A., Kan Y.W. (1994). Isolation of NF-E2-related factor 2 (Nrf2), a NF-E2-like basic leucine zipper transcriptional activator that binds to the tandem NF-E2/AP1 repeat of the beta-globin locus control region. Proc. Natl. Acad. Sci. USA.

[70] Hayes J.D., Dinkova-Kostova A.T. (2014). The Nrf2 regulatory network provides an interface between redox and intermediary metabolism. Trends Biochem. Sci..

[71] Wu K.C., Cui J.Y., Klaassen C.D. (2012). Effect of graded Nrf2 activation on phase-I and -II drug metabolizing enzymes and transporters in mouse liver. PLoS ONE.

[72] Wakabayashi N., Itoh K., Wakabayashi J., Motohashi H., Noda S., Takahashi S., Imakado S., Kotsuji T., Otsuka F., Roop D.R. (2003). Keap1-null mutation leads to postnatal lethality due to constitutive Nrf2 activation. Nat. Genet..

[73] Wu K.C., Cui J.Y., Klaassen C.D. (2011). Beneficial role of Nrf2 in regulating NADPH generation and consumption. Toxicol. Sci..

[74] Reisman S.A., Yeager R.L., Yamamoto M., Klaassen C.D. (2009). Increased Nrf2 activation in livers from Keap1-knockdown mice increases expression of cytoprotective genes that detoxify electrophiles more than those that detoxify reactive oxygen species. Toxicol. Sci..

[75] He F., Antonucci L., Yamachika S., Zhang Z., Taniguchi K., Umemura A., Hatzivassiliou G., Roose-Girma M., Reina-Campos M., Duran A. (2020). NRF2 activates growth factor genes and downstream AKT signaling to induce mouse and human hepatomegaly. J. Hepatol..

[76] Hanahan D., Weinberg R.A. (2011). Hallmarks of cancer: The next generation. Cell.

[77] Fu J., Xiong Z., Huang C., Li J., Yang W., Han Y., Paiboonrungruan C., Major M.B., Chen K.N., Kang X. (2019). Hyperactivity of the transcription factor Nrf2 causes metabolic reprogramming in mouse esophagus. J. Biol. Chem..

[78] Rojo de la Vega M., Chapman E., Zhang D.D. (2018). NRF2 and the Hallmarks of Cancer. Cancer Cell.

[79] Zhang Y., Chen K., Sloan S.A., Bennett M.L., Scholze A.R., O’Keeffe S., Phatnani H.P., Guarnieri P., Caneda C., Ruderisch N. (2014). An RNA-sequencing transcriptome and splicing database of glia, neurons, and vascular cells of the cerebral cortex. J. Neurosci..

[80] Cuadrado A., Rojo A.I., Wells G., Hayes J.D., Cousin S.P., Rumsey W.L., Attucks O.C., Franklin S., Levonen A.L., Kensler T.W. (2019). Therapeutic targeting of the NRF2 and KEAP1 partnership in chronic diseases. Nat. Rev. Drug Discov..

[81] Zahra K., Dey T., Ashish, Mishra S.P., Pandey U. (2020). Pyruvate Kinase M2 and Cancer: The Role of PKM2 in Promoting Tumorigenesis. Front. Oncol..

[82] Yates M.S., Tran Q.T., Dolan P.M., Osburn W.O., Shin S., McCulloch C.C., Silkworth J.B., Taguchi K., Yamamoto M., Williams C.R. (2009). Genetic versus chemoprotective activation of Nrf2 signaling: Overlapping yet distinct gene expression profiles between Keap1 knockout and triterpenoid-treated mice. Carcinogenesis.

[83] Uruno A., Furusawa Y., Yagishita Y., Fukutomi T., Muramatsu H., Negishi T., Sugawara A., Kensler T.W., Yamamoto M. (2013). The Keap1-Nrf2 system prevents onset of diabetes mellitus. Mol. Cell. Biol..

[84] Uruno A., Yagishita Y., Katsuoka F., Kitajima Y., Nunomiya A., Nagatomi R., Pi J., Biswal S.S., Yamamoto M. (2016). Nrf2-Mediated Regulation of Skeletal Muscle Glycogen Metabolism. Mol. Cell. Biol..

[85] Singh A., Happel C., Manna S.K., Acquaah-Mensah G., Carrerero J., Kumar S., Nasipuri P., Krausz K.W., Wakabayashi N., Dewi R. (2013). Transcription factor NRF2 regulates miR-1 and miR-206 to drive tumorigenesis. J. Clin. Investig..

[86] Lunt S.Y., Vander Heiden M.G. (2011). Aerobic glycolysis: Meeting the metabolic requirements of cell proliferation. Annu. Rev. Cell Dev. Biol..

[87] Romero R., Sayin V.I., Davidson S.M., Bauer M.R., Singh S.X., LeBoeuf S.E., Karakousi T.R., Ellis D.C., Bhutkar A., Sanchez-Rivera F.J. (2017). Keap1 loss promotes Kras-driven lung cancer and results in dependence on glutaminolysis. Nat. Med..

[88] DeNicola G.M., Chen P.H., Mullarky E., Sudderth J.A., Hu Z., Wu D., Tang H., Xie Y., Asara J.M., Huffman K.E. (2015). NRF2 regulates serine biosynthesis in non-small cell lung cancer. Nat. Genet..

[89] Sayin V.I., LeBoeuf S.E., Singh S.X., Davidson S.M., Biancur D., Guzelhan B.S., Alvarez S.W., Wu W.L., Karakousi T.R., Zavitsanou A.M. (2017). Activation of the NRF2 antioxidant program generates an imbalance in central carbon metabolism in cancer. Elife.

[90] Tanaka Y., Ikeda T., Yamamoto K., Ogawa H., Kamisako T. (2012). Dysregulated expression of fatty acid oxidation enzymes and iron-regulatory genes in livers of Nrf2-null mice. J. Gastroenterol. Hepatol..

[91] Pi J., Leung L., Xue P., Wang W., Hou Y., Liu D., Yehuda-Shnaidman E., Lee C., Lau J., Kurtz T.W. (2010). Deficiency in the nuclear factor E2-related factor-2 transcription factor results in impaired adipogenesis and protects against diet-induced obesity. J. Biol. Chem..

[92] Maruyama A., Tsukamoto S., Nishikawa K., Yoshida A., Harada N., Motojima K., Ishii T., Nakane A., Yamamoto M., Itoh K. (2008). Nrf2 regulates the alternative first exons of CD36 in macrophages through specific antioxidant response elements. Arch. Biochem. Biophys..

[93] Harada N., Kanayama M., Maruyama A., Yoshida A., Tazumi K., Hosoya T., Mimura J., Toki T., Maher J.M., Yamamoto M. (2011). Nrf2 regulates ferroportin 1-mediated iron efflux and counteracts lipopolysaccharide-induced ferroportin 1 mRNA suppression in macrophages. Arch. Biochem. Biophys..

[94] Kerins M.J., Ooi A. (2018). The Roles of NRF2 in Modulating Cellular Iron Homeostasis. Antioxid. Redox Signal..

[95] Wang S., Kaufman R.J. (2012). The impact of the unfolded protein response on human disease. J. Cell Biol..

[96] Pajares M., Cuadrado A., Rojo A.I. (2017). Modulation of proteostasis by transcription factor NRF2 and impact in neurodegenerative diseases. Redox Biol..

[97] Glover-Cutter K.M., Lin S., Blackwell T.K. (2013). Integration of the unfolded protein and oxidative stress responses through SKN-1/Nrf. PLoS Genet..

[98] Zhu Y.P., Zheng Z., Hu S., Ru X., Fan Z., Qiu L., Zhang Y. (2019). Unification of Opposites between Two Antioxidant Transcription Factors Nrf1 and Nrf2 in Mediating Distinct Cellular Responses to the Endoplasmic Reticulum Stressor Tunicamycin. Antioxidants (Basel).

[99] Harding H.P., Zhang Y., Zeng H., Novoa I., Lu P.D., Calfon M., Sadri N., Yun C., Popko B., Paules R. (2003). An integrated stress response regulates amino acid metabolism and resistance to oxidative stress. Mol. Cell.

[100] Mukaigasa K., Tsujita T., Nguyen V.T., Li L., Yagi H., Fuse Y., Nakajima-Takagi Y., Kato K., Yamamoto M., Kobayashi M. (2018). Nrf2 activation attenuates genetic endoplasmic reticulum stress induced by a mutation in the phosphomannomutase 2 gene in zebrafish. Proc. Natl. Acad. Sci. USA.

[101] Kwak M.K., Wakabayashi N., Greenlaw J.L., Yamamoto M., Kensler T.W. (2003). Antioxidants enhance mammalian proteasome expression through the Keap1-Nrf2 signaling pathway. Mol. Cell. Biol..

[102] Pickering A.M., Linder R.A., Zhang H., Forman H.J., Davies K.J. (2012). Nrf2-dependent induction of proteasome and Pa28alphabeta regulator are required for adaptation to oxidative stress. J. Biol. Chem..

[103] Arlt A., Bauer I., Schafmayer C., Tepel J., Muerkoster S.S., Brosch M., Roder C., Kalthoff H., Hampe J., Moyer M.P. (2009). Increased proteasome subunit protein expression and proteasome activity in colon cancer relate to an enhanced activation of nuclear factor E2-related factor 2 (Nrf2). Oncogene.

[104] Kapeta S., Chondrogianni N., Gonos E.S. (2010). Nuclear erythroid factor 2-mediated proteasome activation delays senescence in human fibroblasts. J. Biol. Chem..

[105] Jang J., Wang Y., Kim H.S., Lalli M.A., Kosik K.S. (2014). Nrf2, a regulator of the proteasome, controls self-renewal and pluripotency in human embryonic stem cells. Stem Cells.

[106] Li B., Fu J., Chen P., Ge X., Li Y., Kuiatse I., Wang H., Wang H., Zhang X., Orlowski R.Z. (2015). The Nuclear Factor (Erythroid-derived 2)-like 2 and Proteasome Maturation Protein Axis Mediate Bortezomib Resistance in Multiple Myeloma. J. Biol. Chem..

[107] Rushworth S.A., Bowles K.M., MacEwan D.J. (2011). High basal nuclear levels of Nrf2 in acute myeloid leukemia reduces sensitivity to proteasome inhibitors. Cancer Res..

[108] Clarke R., Cook K.L., Hu R., Facey C.O., Tavassoly I., Schwartz J.L., Baumann W.T., Tyson J.J., Xuan J., Wang Y. (2012). Endoplasmic reticulum stress, the unfolded protein response, autophagy, and the integrated regulation of breast cancer cell fate. Cancer Res..

[109] Pajares M., Jimenez-Moreno N., Garcia-Yague A.J., Escoll M., De Ceballos M.L., Van Leuven F., Rabano A., Yamamoto M., Rojo A.I., Cuadrado A. (2016). Transcription factor NFE2L2/NRF2 is a regulator of macroautophagy genes. Autophagy.

[110] Jain A., Lamark T., Sjottem E., Larsen K.B., Awuh J.A., Overvatn A., McMahon M., Hayes J.D., Johansen T. (2010). p62/SQSTM1 is a target gene for transcription factor NRF2 and creates a positive feedback loop by inducing antioxidant response element-driven gene transcription. J. Biol. Chem..

[111] Holmstrom K.M., Baird L., Zhang Y., Hargreaves I., Chalasani A., Land J.M., Stanyer L., Yamamoto M., Dinkova-Kostova A.T., Abramov A.Y. (2013). Nrf2 impacts cellular bioenergetics by controlling substrate availability for mitochondrial respiration. Biol. Open.

[112] Ludtmann M.H., Angelova P.R., Zhang Y., Abramov A.Y., Dinkova-Kostova A.T. (2014). Nrf2 affects the efficiency of mitochondrial fatty acid oxidation. Biochem. J..

[113] Anedda A., Lopez-Bernardo E., Acosta-Iborra B., Saadeh Suleiman M., Landazuri M.O., Cadenas S. (2013). The transcription factor Nrf2 promotes survival by enhancing the expression of uncoupling protein 3 under conditions of oxidative stress. Free Radic. Biol. Med..

[114] Hu Q., Ren J., Li G., Wu J., Wu X., Wang G., Gu G., Ren H., Hong Z., Li J. (2018). The mitochondrially targeted antioxidant MitoQ protects the intestinal barrier by ameliorating mitochondrial DNA damage via the Nrf2/ARE signaling pathway. Cell Death Dis..

[115] Piantadosi C.A., Carraway M.S., Babiker A., Suliman H.B. (2008). Heme oxygenase-1 regulates cardiac mitochondrial biogenesis via Nrf2-mediated transcriptional control of nuclear respiratory factor-1. Circ. Res..

[116] Merry T.L., Ristow M. (2016). Nuclear factor erythroid-derived 2-like 2 (NFE2L2, Nrf2) mediates exercise-induced mitochondrial biogenesis and the anti-oxidant response in mice. J. Physiol..

[117] Aguiar A.S., Duzzioni M., Remor A.P., Tristao F.S., Matheus F.C., Raisman-Vozari R., Latini A., Prediger R.D. (2016). Moderate-Intensity Physical Exercise Protects Against Experimental 6-Hydroxydopamine-Induced Hemiparkinsonism Through Nrf2-Antioxidant Response Element Pathway. Neurochem. Res..

[118] Gureev A.P., Shaforostova E.A., Popov V.N. (2019). Regulation of Mitochondrial Biogenesis as a Way for Active Longevity: Interaction Between the Nrf2 and PGC-1alpha Signaling Pathways. Front. Genet..

[119] Bagger F.O., Sasivarevic D., Sohi S.H., Laursen L.G., Pundhir S., Sonderby C.K., Winther O., Rapin N., Porse B.T. (2016). BloodSpot: A database of gene expression profiles and transcriptional programs for healthy and malignant haematopoiesis. Nucleic Acids Res..

[120] Thimmulappa R.K., Lee H., Rangasamy T., Reddy S.P., Yamamoto M., Kensler T.W., Biswal S. (2006). Nrf2 is a critical regulator of the innate immune response and survival during experimental sepsis. J. Clin. Investig..

[121] Iizuka T., Ishii Y., Itoh K., Kiwamoto T., Kimura T., Matsuno Y., Morishima Y., Hegab A.E., Homma S., Nomura A. (2005). Nrf2-deficient mice are highly susceptible to cigarette smoke-induced emphysema. Genes Cells.

[122] Tang W., Jiang Y.F., Ponnusamy M., Diallo M. (2014). Role of Nrf2 in chronic liver disease. World J. Gastroenterol..

[123] Yoh K., Itoh K., Enomoto A., Hirayama A., Yamaguchi N., Kobayashi M., Morito N., Koyama A., Yamamoto M., Takahashi S. (2001). Nrf2-deficient female mice develop lupus-like autoimmune nephritis. Kidney Int..

[124] Ma Q., Battelli L., Hubbs A.F. (2006). Multiorgan autoimmune inflammation, enhanced lymphoproliferation, and impaired homeostasis of reactive oxygen species in mice lacking the antioxidant-activated transcription factor Nrf2. Am. J. Pathol..

[125] Osburn W.O., Yates M.S., Dolan P.D., Chen S., Liby K.T., Sporn M.B., Taguchi K., Yamamoto M., Kensler T.W. (2008). Genetic or pharmacologic amplification of nrf2 signaling inhibits acute inflammatory liver injury in mice. Toxicol. Sci..

[126] Innamorato N.G., Rojo A.I., Garcia-Yague A.J., Yamamoto M., De Ceballos M.L., Cuadrado A. (2008). The transcription factor Nrf2 is a therapeutic target against brain inflammation. J. Immunol..

[127] Kobayashi E.H., Suzuki T., Funayama R., Nagashima T., Hayashi M., Sekine H., Tanaka N., Moriguchi T., Motohashi H., Nakayama K. (2016). Nrf2 suppresses macrophage inflammatory response by blocking proinflammatory cytokine transcription. Nat. Commun..

[128] Hoetzenecker W., Echtenacher B., Guenova E., Hoetzenecker K., Woelbing F., Bruck J., Teske A., Valtcheva N., Fuchs K., Kneilling M. (2011). ROS-induced ATF3 causes susceptibility to secondary infections during sepsis-associated immunosuppression. Nat. Med..

[129] Bambouskova M., Gorvel L., Lampropoulou V., Sergushichev A., Loginicheva E., Johnson K., Korenfeld D., Mathyer M.E., Kim H., Huang L.H. (2018). Electrophilic properties of itaconate and derivatives regulate the IkappaBzeta-ATF3 inflammatory axis. Nature.

[130] Wruck C.J., Streetz K., Pavic G., Gotz M.E., Tohidnezhad M., Brandenburg L.O., Varoga D., Eickelberg O., Herdegen T., Trautwein C. (2011). Nrf2 induces interleukin-6 (IL-6) expression via an antioxidant response element within the IL-6 promoter. J. Biol. Chem..

[131] Burgener A.V., Bantug G.R., Meyer B.J., Higgins R., Ghosh A., Bignucolo O., Ma E.H., Loeliger J., Unterstab G., Geigges M. (2019). SDHA gain-of-function engages inflammatory mitochondrial retrograde signaling via KEAP1-Nrf2. Nat. Immunol..

[132] Harvey C.J., Thimmulappa R.K., Sethi S., Kong X., Yarmus L., Brown R.H., Feller-Kopman D., Wise R., Biswal S. (2011). Targeting Nrf2 signaling improves bacterial clearance by alveolar macrophages in patients with COPD and in a mouse model. Sci. Transl. Med..

[133] Olagnier D., Brandtoft A.M., Gunderstofte C., Villadsen N.L., Krapp C., Thielke A.L., Laustsen A., Peri S., Hansen A.L., Bonefeld L. (2018). Nrf2 negatively regulates STING indicating a link between antiviral sensing and metabolic reprogramming. Nat. Commun..

[134] Page A., Volchkova V.A., Reid S.P., Mateo M., Bagnaud-Baule A., Nemirov K., Shurtleff A.C., Lawrence P., Reynard O., Ottmann M. (2014). Marburgvirus hijacks nrf2-dependent pathway by targeting nrf2-negative regulator keap1. Cell Rep..

[135] Saddawi-Konefka R., Seelige R., Gross E.T., Levy E., Searles S.C., Washington A., Santosa E.K., Liu B., O’Sullivan T.E., Harismendy O. (2016). Nrf2 Induces IL-17D to Mediate Tumor and Virus Surveillance. Cell Rep..

[136] Swann J.B., Smyth M.J. (2007). Immune surveillance of tumors. J. Clin. Investig..

[137] Hou J., Zhang H., Sun B., Karin M. (2020). The immunobiology of hepatocellular carcinoma in humans and mice: Basic concepts and therapeutic implications. J. Hepatol..

[138] Ohl K., Fragoulis A., Klemm P., Baumeister J., Klock W., Verjans E., Boll S., Mollmann J., Lehrke M., Costa I. (2018). Nrf2 Is a Central Regulator of Metabolic Reprogramming of Myeloid-Derived Suppressor Cells in Steady State and Sepsis. Front. Immunol..

[139] Nagaraj S., Gupta K., Pisarev V., Kinarsky L., Sherman S., Kang L., Herber D.L., Schneck J., Gabrilovich D.I. (2007). Altered recognition of antigen is a mechanism of CD8+ T cell tolerance in cancer. Nat. Med..

[140] Hiramoto K., Satoh H., Suzuki T., Moriguchi T., Pi J., Shimosegawa T., Yamamoto M. (2014). Myeloid lineage-specific deletion of antioxidant system enhances tumor metastasis. Cancer Prev. Res. (Phila.).

[141] Sha L.K., Sha W., Kuchler L., Daiber A., Giegerich A.K., Weigert A., Knape T., Snodgrass R., Schroder K., Brandes R.P. (2015). Loss of Nrf2 in bone marrow-derived macrophages impairs antigen-driven CD8(+) T cell function by limiting GSH and Cys availability. Free Radic. Biol. Med..

[142] Suzuki T., Murakami S., Biswal S.S., Sakaguchi S., Harigae H., Yamamoto M., Motohashi H. (2017). Systemic Activation of NRF2 Alleviates Lethal Autoimmune Inflammation in Scurfy Mice. Mol. Cell. Biol..

[143] Rockwell C.E., Zhang M., Fields P.E., Klaassen C.D. (2012). Th2 skewing by activation of Nrf2 in CD4(+) T cells. J. Immunol..

[144] Yarosz E.L., Chang C.H. (2018). The Role of Reactive Oxygen Species in Regulating T Cell-mediated Immunity and Disease. Immune Netw..

[145] Aw Yeang H.X., Hamdam J.M., Al-Huseini L.M., Sethu S., Djouhri L., Walsh J., Kitteringham N., Park B.K., Goldring C.E., Sathish J.G. (2012). Loss of transcription factor nuclear factor-erythroid 2 (NF-E2) p45-related factor-2 (Nrf2) leads to dysregulation of immune functions, redox homeostasis, and intracellular signaling in dendritic cells. J. Biol. Chem..

[146] Zhao M., Chen H., Ding Q., Xu X., Yu B., Huang Z. (2016). Nuclear Factor Erythroid 2-related Factor 2 Deficiency Exacerbates Lupus Nephritis in B6/lpr mice by Regulating Th17 Cell Function. Sci. Rep..

[147] Chen X., Su W., Wan T., Yu J., Zhu W., Tang F., Liu G., Olsen N., Liang D., Zheng S.G. (2017). Sodium butyrate regulates Th17/Treg cell balance to ameliorate uveitis via the Nrf2/HO-1 pathway. Biochem. Pharmacol..

[148] Noel S., Lee S.A., Sadasivam M., Hamad A.R.A., Rabb H. (2018). KEAP1 Editing Using CRISPR/Cas9 for Therapeutic NRF2 Activation in Primary Human T Lymphocytes. J. Immunol..

[149] Maj T., Wang W., Crespo J., Zhang H., Wang W., Wei S., Zhao L., Vatan L., Shao I., Szeliga W. (2017). Oxidative stress controls regulatory T cell apoptosis and suppressor activity and PD-L1-blockade resistance in tumor. Nat. Immunol..

